# Efficacy and safety of stem cell mobilization with etoposide +cytarabine plus G-CSF in poor mobilizers with relapsed or refractory lymphoma

**DOI:** 10.3389/fimmu.2024.1439253

**Published:** 2024-07-18

**Authors:** Zhijuan Zhu, Xiaofan Li, Xiaohong Yuan, Xianling Chen, Ting Lin, Xiangli Guo, Nainong Li

**Affiliations:** ^1^ Hematopoietic Stem Cell Transplantation Center, Fujian Institute of Hematology, Fujian Provincial Key Laboratory on Hematology, Department of Hematology, Fujian Medical University Union Hospital, Fuzhou, China; ^2^ Translational Medicine Center on Hematology, Fujian Medical University, Fuzhou, China

**Keywords:** etoposide, cytarabine, stem cell mobilization, lymphoma, poor mobilizers

## Abstract

**Background:**

Autologous stem cell transplantation (ASCT) is a potentially curative strategy for relapse or refractory(r/r) aggressive lymphoma. However, a proportion of lymphoma patients who are at high risk of mobilization failure fail to mobilize stem cells and cannot proceed to ASCT. The aim of this study is to explore the efficacy and safety of Etoposide combined with Cytarabine (EA) plus G-CSF mobilization in poor mobilizers (PMs) with r/r aggressive lymphoma.

**Methods:**

This retrospective study analyzed the outcomes of chemo-mobilization based on EA (Etoposide 0.1 g/m2, qd d1~3; AraC 0.5 g/m2, q12h d1~3) in 98 patients with r/r aggressive lymphoma. Of these, 39 patients met the criteria for predicted PMs as proposed by the Gruppo Italiano Trapianto di Midollo Osseo working group.

**Results:**

Of the 39 PMs, 38(97.4%) patents harvested adequate mobilization (≥2×10^6^ CD34+ cells/kg), while 31(79.5%) patients achieved optimal mobilization (≥5×10^6^ CD34+ cells/kg). Overall, the mean number of CD34+ cells/kg collected was 17.99(range: 1.08~83.07) ×10^6^ with an average of 1.4 apheresis sessions, and the number was 15.86(range: 0.37~83.07) ×10^6^ for the first apheresis, respectively. A single apheresis procedure was sufficient to reach the target yield of adequate mobilization in 35(89.7%) PMs, while 76.9% of PMs achieved optimal collection within two apheresis sessions. We observed acceptable hematological toxicity and antibiotic usage exposure in 26 patients with a mean duration of 3.6 days. No grade 4 infection or mobilization-related mortality was recorded. Most patients underwent ASCT and achieved successful hematopoietic recovery with prompt engraftment duration, except for one NK/T-cell lymphoma patient who succumbed to severe septicemia after receiving conditioning chemotherapy.

**Conclusion:**

Our findings indicate that EA plus G-CSF is an effective and tolerable CD34+ stem cell mobilization strategy for patients with r/r lymphoma, including those predicted to be PMs. This regimen could be an option for patients with r/r lymphoma, particularly those undergoing mobilization for salvage ASCT therapy.

## 1 Background

Autologous stem cell transplantation (ASCT) is a potentially curative strategy for relapse or refractory (r/r) aggressive non-Hodgkin lymphoma (NHL) and Hodgkin lymphoma (HL) (1–4). Successful peripheral blood stem cell (PBSC) mobilization is crucial for ASCT, ensuring prompt hematopoietic recovery, with a minimum adequate total CD34+ yield of 2x10^6^ cells/kg and an optimal target of 5x10^6^ cells/kg (5, 6). However, lymphoma patients fail to mobilize sufficient PBSCs more often than multiple myeloma (MM) patient (7–10). Several factors were identified to predict poor mobilization, including old age, prior extensive radiotherapy, long-term antecedent chemotherapy and disease status (11). Despite advancements in commonly used chemo-mobilization such as CY, DHAP and GDP, up to 40% of lymphoma patients fail to mobilize adequate PBSCs, hindering their ability to proceed to ASCT (12–15). The failure rate is even higher in patients at high risk of mobilization failure (5, 16, 17). CXCR4 antagonists, such as plerixafor and motixafortide, have garnered attention for its efficacy in PBSC mobilization for ‘proven’ or ‘predicted poor mobilizers’ (10, 18–21), but its high cost limits its availability in many countries. Therefore, the optimal front-line mobilization strategy remains a topic of debate in r/r aggressive lymphoma, especially those defined as poor mobilizers.

Recently, several studies have explored single high dose etoposide or intermediate-dose cytarabine as chemo-mobilization regimens in poor mobilizing lymphoma patients, yielding promising adequate collection (7, 22–24). However, only up to 60% lymphoma patients achieved optimal mobilization. In addition, the utilization of high doses of etoposide carries the risk of developing a secondary tumor. Our group previously reported the high efficacy of autologous PBSC mobilization using etoposide and cytarabine (EA) plus G-CSF in MM (25). In this study, all patients mobilized successfully, with 94.5% achieving optimal collection and a median overall collected CD34+ cell count of 28.23×10^6^/kg. However, whether this novel EA protocol can achieve comparable outcomes in r/r aggressive lymphoma, especially in those at high risk of mobilization failure, remains unknown. Here we retrospectively studied the efficacy and safety of CD34+ PBSC mobilization using EA followed by G-CSF in 98 r/r lymphoma patients, including 39 predicted poor mobilizers (PMs).

## 2 Materials and method

### 2.1 Patients

All patients with r/r lymphoma who underwent ASCT using EA plus G-CSF as the first-line PBSC mobilization regimen at Fujian Medical University Union Hospital between April 2013 and March 2022 were eligible for study enrollment. Patients were defined as predicted PMs if they met the criteria proposed by the Gruppo Italiano Trapianto di Midollo Osseo working group (1): they failed a previous collection attempt (not otherwise specified); (2) they previously received extensive radiotherapy to marrow bearing tissue or full courses of therapy affecting SC mobilization; and (3) they met two of the following criteria: advanced disease (>2 lines of chemotherapy), refractory disease, extensive BM involvement or cellularity <30% at the time of mobilization; age> 65 years (11). Additionally, extensive radiotherapy referred to patients received large-field extensive radiotherapy to marrow bearing tissue, with total doses exceeding 30 Gy. Full courses of therapy included previous exposure to fludarabine ≥1 cycles, melphalan ≥2 cycles, or other therapies potentially affecting stem cell mobilization (when the dose-intensity or composition of the cytostatic regimens could not be taken into account, previous chemocherapy >6 cycles). Patients undergoing mobilization for salvage ASCT therapy were also included (patients who did not achieve partial remission or a better treatment response before PBSC mobilization). The treatment response for lymphoma was determined as previously described (26). The study protocol was approved by the ethics committee of Union Hospital, Fujian Medical University, and informed consents were obtained from all patients before the study.

### 2.2 Mobilization regimens and leukapheresis

The administration of EA mobilization regimens and leukapheresis protocols was previously reported by our group (25). All patients received EA (etoposide 100 mg/m^2^, qd d1~3; cytarabine 0.5 g/m^2^, q12h d1~3) and G-CSF (5 µg/kg/day) from d5 until the last day of apheresis. Etoposide dosage was reduced to 50mg/m^2^ if patients suffered from chronic kidney disease stage ≥3. PBSC collection was initiated based on the CD34+ cell or hematopoietic progenitor cell (HPC) count in the peripheral blood. The primary apheresis target in our center was ≥4×10^6^/kg and the maximum sessions of leukapheresis was 3. Due to the improvement in the administration of the EA protocol, patients admitted to the hospital for the entire mobilization period from 2013 to 2016. Since 2017, patients were discharged after 3 days of chemotherapy usage, and then they received daily subcutaneous injections of G-CSF for 6 days in the outpatient clinic. All patients were asked to re-hospitalize on d10 until the completion of leukapheresis. Importantly, no prophylactic antibiotics were administered during neutropenia.

### 2.3 ASCT and engraftment

The conditioning regimen used for ASCT consisted of either BEAM (carmustine 150 mg/m^2^ qd d-6~d-7; cytarabine 200mg/m2 q12h d-5~d-2; etoposide 200mg/m2 qd d-5~d-2; melphalan 140mg/m2 qd d-1) or CBV (carmustine 150 mg/m2 qd d-8~d-10;etoposide 15 mg/kg qd d-4~d-7; cyclophosphamide 100mg/kg qd d-2) according to physician’s consideration. When carmustine was unavailable, oral simustine (250mg/m^2^ in BEAM, and 400mg/m^2^ in CBV) was used as an alternative in our institution. For patients with CD20 positive lymphoma, rituximab (375mg/m2, qd, d+1, d+8) was added to the regimen. G-CSF (5 µg/kg/day) was administered starting 5 days after PBSC reinfusion and continued until neutrophil engraftment. Neutrophil and platelet engraftment were defined according to the guidelines on post-transplant essential data from the Center for International Blood and Marrow Transplantation Research (CIBMTR).

### 2.4 Outcome and definitions

As previously reported, optimal mobilization was defined as mobilized CD34+cell ≥5×10^6/^kg, and adequate mobilization was defined as mobilized CD34+ cell ≥2×10^6^/kg (5). The primary endpoint was to determine the proportion of patients achieving adequate and optimal mobilization. Secondary endpoints included the mean number of apheresis procedures performed, the timing of apheresis initiation, adverse events and hospitalization days associated with EA administration. Adverse events included hematologic toxicity, were graded according to the Common Terminology Criteria for Adverse Events version 4.0 (27).

### 2.5 Statistical analysis

The data analysis was conducted utilizing the IPSS version 25.0(SPSS, Chicago, IL, USA). Normality of the data was assessed through the kolmogorov-smirnov Z test. Categorical variables were compared using the χ2 test or Fisher exact test, while differences in quantitative variables between groups were examined using the Mann-Whitney U test. Statistical significance was set at a two-sided p-value < 0.05 for all analyses.

## 3 Results

### 3.1 Characteristics of patients

A total of 98 patients diagnosed with r/r lymphoma received EA as chemo-mobilization regimen, of which 39 patients were categorized as PMs and enrolled in the study. Totally, the median age of the PM cohort was 40 years (range: 8-63 years), with 26(66.7%) being males. Only 6(15.4%) patients weighed over 75kg. Upon initial diagnosis, 16(41.0%) patients had IPI scores ≥3. Concerning histological subtypes, 5(12.8%) patients were HL and 34(87.2%) patients were NHL (including 26 NHL-B, 5 NHL-T and 3 NHL-NK). Prior to mobilization, 10(25.6%) patients had achieved complete response, while 14(35.9%) patients were either in a stable or progressive disease stage. Additionally, 37(94.8%) patients received two or more lines of preceding chemotherapy, and the median number of prior chemotherapy cycles was 10(range: 3-17). Furthermore, the median interval from diagnosis to mobilization was 15 months. Summary of patient characteristics is presented in **Table 1**.

**Table 1:** Patients’ baseline characteristics.

Variables	Total	PMs
	(*n*=98)	(*n*=39)
Age, years, median(range)	45(8~63)	40(8~63)
Sex, male, *n*(%)	63(64.3%)	26(66.7%)
Weight,kg,median(range)	60(22~86)	60(22~85)
IPI score≥3, *n*(%)	36(36.7%)	16(41.0%)
Diagnosis, *n*(%)		
HL	8(8.2%)	5(12.8%)
NHL-B	67(68.4%)	26(66.7%)
NHL-T	9(9.2%)	5(12.8%)
NHL-NKT	14(14.3%	3(7.7%)
Month from diagnosis to mobilization, median (range)	7(3~94)	15(3~59)
Lines of preceding therapy, *n*(%)		
1	41(41.8%)	2(5.1%)
2	37(37.8%)	21(53.8%)
≥3	20(20.4%)	16(41.0%)
Cycles of preceding therapy, median(range)	6(3~17)	10(3~17)
Response to prior treatment, *n*(%)		
CR	55(56.7%)	10(25.6%)
PR	25(25.8%)	15(38.5%)
SD	5(5.2%)	4(10.3%)
PD	12(12.4%)	10(25.6%)

HL, hodgkin lymphoma; NHL,non-hodgkin’s lymphoma; CR, Complete response; PR, partial remission; SD, stable disease; PD, progressive disease; PMs, predicted poor mobilizers.

### 3.2 Efficacy of PBSC mobilization

CD34+ cells were uniformly detected in all PBSC samples collected from the patients, utilizing flow cytometry. Among the PM cohort, the median peak number of circulating CD34+ cells was 148.0/ul, while the median number of overall and first day collected CD34+ cells was determined to be 17.99×10^6^/kg and 15.86×10^6^/kg, respectively. Three patients failed to achieve ≥20/μL circulating CD34+ cells before the first apheresis, with their counts ranging between 10-20/μL. Remarkably, of the 39 PMs, only one patient failed to target adequate mobilization, while 31(79.5%) achieved optimal mobilization through a median of 1 apheresis sessions. Notably, a single apheresis was sufficient to target an adequate number of CD34+ cells for ASCT in 35(89.7%) patients with r/r lymphoma, with 76.9%(30/39) predicted PM patients achieving optimal collection within two apheresis sessions. Compared to patients who achieved partial remission or a better response before mobilization, the median number of overall CD34+ cells collected was significantly lower in those with a worse response (6.65 vs. 22.12×10^6^/kg, *p*=0.004). Further analysis was performed to compare the mobilization efficacy between the PMs and the other 59 lymphoma patients who were not categorized as PMs, no significant differences were found between the two groups concerning the rates of adequate (97.4% vs. 98.3%) or optimal(79.5% vs. 84.7%) mobilization. Additionally, the median interval from mobilization to collection was found to be 15 days overall. Subsequent improvement in mobilization strategy led to a reduction in the median length of hospitalization, from 15 days (range: 13-19 days) for the initial 16 patients during 2013-2016 to 6 days (range: 3-11 days) for the subsequent 23 patients after 2017. These findings are summarized in **Table 2**.

**Table 2:** Efficacy of mobilization.

Variables	Total	PMs
	(*n*=98)	(*n*=39)
Total CD34+ cells collected, ×10^6^/kg, mean (range)	22.39(1.08~138.96)	17.99(1.08~83.07)
First day CD34+ cells collected, ×10^6^/kg, mean (range)	20.63(0.1~138.96)	15.86(0.37~83.07)
Total Collection target, *n* (%)		
≥2×10^6^ CD34+ cells/kg	96(98.0%)	38(97.4%)
≥5×10^6^ CD34+ cells/kg	81(82.7%)	31(79.5%)
Day 1 apheresis day target, *n* (%)		
≥2×10^6^ CD34+ cells/kg	85(86.7%)	35(89.7%)
≥5×10^6^ CD34+ cells/kg	71(72.4%)	27(69.2%)
Day 1and 2 apheresis target, *n* (%)		
≥2×10^6^ CD34+ cells/kg	92(93.9%)	35(89.7%)
≥5×10^6^ CD34+ cells/kg	79(80.6%)	30(76.9%)
Days of leukapheresis initiation, median(range)	14(11~19)	15(11~19)
Sessions of apheresis, median(range)	1(1~3)	1(1~3)
Sessions of apheresis, *n* (%)		
1 session	68(69.4%)	27(69.2%)
2 sessions	23(23.5%)	8(20.5%)
3 sessions	7(7.1%)	4(10.3%)

PMs, predicted poor mobilizers.

### 3.3 Toxicity

The predominant complication observed in our study was bone marrow suppression. However, no instances of bleeding or mortality were recorded. Overall, all PMs experienced grade 4 neutropenia, and 37(94.9%) patients experienced grade 4 thrombocytopenia. The median duration of grade 4 thrombocytopenia was 3 days (range: 0-8 days), requiring minimal platelet transfusions (median 1.9, range: 0-4.4) prior to apheresis. Although 38(97.4%) PM patients required platelet infusions, only 4(10.3%) patients needed RBC infusions during mobilization. Overall, 26 (66.7%) PM patients required antibiotic therapy for grade 2-3 infections, with no occurrences of grade 4 infections during mobilization. 24(61.5%) episode of febrile neutropenia were observed. The most common infections were respiratory tract infections (20.5%) and febrile neutropenia of unknown origin (23.1%). Other infection sites included four in the oral cavity mucosa, three perianal, one septicemia, one digestive tract, and one urinary tract. The median duration of antibiotic administration was 4 days (range: 0-10 days). Detailed toxicity data are presented in **Table 3**.

**Table 3:** Common toxicities.

Variables	Total	PMs
	(*n*=98)	(*n*=39)
Days of Grade 4 Neutropenia^a^, median (range)	4(0~9)	4(1~7)
Days of Grade 4 Thrombocytopenia^b^, median (range)	3(0~8)	3(0~8)
Platelet transfusions, mean (range)	1.9(0~4.8)	1.9(0~4.4)
Erythrocyte transfusions, mean (range)	0.2(0~4.0)	0.3(0~4.0)
Infection, *n* (%)	69(70.4%)	26(66.7%)
febrile neutropenia of unknown origin	27(27.6%)	9(23.1%)
respiratory tract infection	24(24.5%)	8(20.5%)
bacteremia	3(3.1%)	1(2.6%)
Others^c^, n(%)	15(15.3%)	8(20.5%)
Days of antibiotics usage, median(range)	4(0~10)	4(0~10)

PMs, predicted poor mobilizers; ^a^ absolute neutrophil count <0.5×109/L; ^b^: platelet count <25.0×109/L; ^c^: include oral cavity infection, perianal infection, digestive tract infection, and urinary tract infection.

### 3.4 Hematologic recovery after ASCT

All PM patients underwent ASCT as planned, and no instances of disease progression were observed during the mobilization-to-transplantation interval (median: 44.5 days). Post PBSC infusion, all PM patients achieved successful hematologic recovery, except for one NK/T-cell lymphoma patient who succumbed to severe septicemia after receiving conditioning chemotherapy. We noted similar neutrophil and platelet engraftment duration (mean: 10.5 vs. 11.5 days). Although platelet transfusions were required by all patients prior to hematologic recovery, only 12 (30.8%) patients necessitated erythrocyte transfusions. Interestingly, lymphoma patients who achieved optimal mobilization experienced faster neutrophil engraftment (median days: 10 vs. 11, p=0.034) and platelet engraftment (median days: 11 vs. 12, p=0.037) compared to those who did not. However, no similar phenomenon was observed in PM patients. Detailed data regarding hematologic reconstitution are summarized in **Table 4**.

**Table 4:** Hematologic recovery after ASCT.

Variable	Total	PMs
	(*n*=96)	(*n*=38)
Months from diagnosis to transplantation,		
median(range)	9(4~95)	17(4~61)
Day of reach, mean (range)		
Neutrophils ≥0.5×10^9^/ul	10.3(7~15)	10.5(8~14)
PLT ≥20×10^9^/ul	12.1(5~47)	11.5(7~23)
Therapeutic doses of platelet transfusions, median(range)	2.0(0.4~8.0)	2(0.6~5.2)
unites of erythrocyte transfusions, median(range)	0(0~6)	0(0~6)

PMs, predicted poor mobilizers.

## 4 Discussion

Poor mobilization of PBSC poses a significant limitation to ASCT in lymphoma (28). Because infusion of PBSC amounts less than 2×10^6^ CD34+ cells/kg can compromise engraftment, and repeated apheresis procedures are associated with increased costs and complications (29–31). Optimal mobilization of autologus CD34+ cells for ASCT has been reported to improve overall survival (OS) and event-free survival in peripheral T-cell lymphoma (32), and higher graft CD34+ cell counts have been associated with better OS in patients with diffuse large B-cell lymphoma (33). Various strategies, such as chemo-mobilization (CM) and the use of CXCR4 antagonists, have been explored to enhance mobilization efficacy in lymphoma, especially those at high risk of mobilization failure. However, commonly used chemo-mobilization regimens or plerixafor have been reported with a 40-61% failure rate in achieving optimal apheresis in poor mobilizers with r/r lymphoma, and the rate was 4-50% in achieving adequate apheresis (19, 22, 24).

In our prior work, we demonstrated EA plus G-CSF as CM was highly effective in patients with MM (25). In the present study, we evaluated the efficacy of this regimen in patients with r/r aggressive lymphoma, particularly those predicted to be poor mobilizers as previously proposed. Consistent with our findings in MM, our results in poor mobilizers with r/r lymphoma were encouraging. Overall, 97.4% of lymphoma patients achieved adequate collection, with 79.5% achieving optimal collection. Remarkably, a single apheresis session was sufficient for collecting an adequate number of CD34+ cells for ASCT in 89.7% (*n*=35/39) of lymphoma patients, a significantly higher rate compared to reported outcomes with plerixafor (56.5%) (10). Furthermore, 76.9%(*n*=30/39) of patients achieved optimal collection within two apheresis sessions. Comparatively, a study using cytarabine plus G-CSF as CM in 28 PMs with MM and lymphoma reported only 61% achieving optimal collection (24), which was lower than our findings. Another study involving 33 lymphoma patients with at least one prior mobilization failure and receiving low dose cytarabine-based (400mg/m2/day×3 days) CM reported a mean CD34+ cell count of 4.69×106 (range: 1.5-6.8×106)/kg (23), whereas in our study, the PMs group had a mean count of 17.99×10^6^/kg. Comparison of mobilization efficacy with published mobilization strategies and our EA protocol in poor mobilizing lymphoma is summarized in **Table 5**.

**Table 5:** Comparison of mobilization efficacy with published mobilization strategies in patients with poor mobilizing lymphoma.

regimens	patient	*n*	CD34+ yield,	failure rates of reaching, %	
	population		×10^6^/kg	2×10^6^/kg	5×10^6^/kg
Cy+G-CSF (24).	HL, NHL, MM	18	4.4	50	61
ID-AraC+G-CSF (22).	HL, NHL	50	6.2	4	40
etoposide+G-CSF (17).	HL, NHL	59	6.33	8.5	42.4
DHAP+G-CSF(22).	HL, NHL	51	4.4	29	61
EAP (33).	HL, NHL	26	4.3	19.2	53.8
Plexixafor (19).	HL, NHL	134	4.4	9	41
EA+G-CSF	HL, NHL	39	18.05	1.7	18.6

ID-AraC, intermediate-dose cytarabine; Cy, cyclophosphamide; DHAP, dexamethasone, cytarbine, and cisplatin; EA, etoposide, cytarabine; EAP, etoposide, cytarabine, pegfilgrastim; MM, multipl myeloma; HL, Hodgkin lymphoma; NHL, non-Hodgkin lymphoma.

Currently, CAR T-cell therapy has shown significant efficacy in treating r/r lymphoma, particularly in B-cell NHL patients with active disease. However, some patients experience delayed hematopoietic recovery following CAR T-cell treatment, leading to severe infections, bleeding, and even mortality. The high quantity of CD34+ cells collected through our EA protocol not only meets the criteria for aggressive lymphoma patients undergoing ASCT for consolidation treatment but also allows for partial cryopreservation of cells. This ensures the availability of cells for future salvage PBSC infusion to avoid prolonged cytopenias following bone marrow suppression treatments, such as CAR-T, upon disease relapse or progression. Our findings revealed no significant difference in mobilization efficacy between PMs and those who were not at high risk of mobilization failure, suggesting that the EA regimen may mitigate the negative impact of high-risk factors on mobilization in lymphoma. Additionally, the absence of previously mobilization-failed patients in our study cohort might have influenced our findings.

Ye et al. recently reported the outcomes of reduced dosage etoposide (75 mg/m2/day, d1-2)+cytarabine (300mg/m2 q12h, day1-2) plus pegfilgrastim in a small cohort of poorly mobilizing MM (*n*=32) and lymphoma (*n*=26) patients (34). Among the 26 lymphoma patients, 21(80.8%) collected adequate amount of CD34+ cells, while only 12(46.2%) achieved optimal mobilization. The total number of CD34+ cells obtained in PMs with lymphoma was lower than that achieved with our protocol (median: 4.3 vs. 12.9×10^6^/kg). Although the dosage reduction of EA was designed to decrease hematologic toxicity, it also impaired PBSC mobilization efficacy in PMs. What’s more, they found one lymphoma patient and one MM patient who did not achieve at least partial remission experienced significant disease progression after collection, preventing them from proceeding to ASCT. None of the patients in our protocol experienced disease progression in lymphoma during a median mobilization-to-transplantation interval of 44.5days. What’s more, in our previous work, we observed 14 of 128 MM patients obtained a deeper response post EA mobilization (25). It is recognized that chemo-mobilization was originally designed to reduce tumor burden while mobilizing PBSC, especially in relapse or refectory patients. Therefore, our protocol may be considered for r/r lymphoma patients who do not achieve partial remission or a better response, to avoid disease progression prior to ASCT. In alignment with published data in lymphoma, the median interval from patients initiating leukapheresis was 15 days post EA administration in lymphoma cases. To optimize efficiency, we initiated peripheral circular CD34+ and HPC detection on the 11th day, benefiting from the relatively predictable apheresis time. One of the main concerns when employing a mobilization protocol is financial burden. Fewer apheresis sessions result in reduced mobilization-related costs and complications. While our center aimed for a primary mobilization goal of 4×10^6^/kg, only 10.3% lymphoma patients required 3 apheresis sessions. Commonly used mobilization regimens based on chemotherapy have been observed with certain grade 3-4 neutropenia or thrombocytopenia (7, 22, 23, 35). In our study, we found more common occurrences of grade 4 hematological adverse events, but no mobilization- related mortality was recorded. Almost all 39 patients necessitated platelet transfusions, with only 4 patients (10.2%) requiring erythrocyte transfusions. Patients deemed at high risk of mobilization failure have been reported to experience more complications. However, in our investigation, PMs exhibited similar durations of grade 4 bone marrow suppression and rates of infection compared to r/r lymphoma patients who were not defined as PMs. Taking the common hematological toxicity into consideration, a moderate reduction in EA dosages for patients at high risk of infection, such as those of advanced age or with a history of severe infections, might further improve the safety profile of EA. It has been reported that high-dose cytotoxic agents utilized in mobilization may lead to delayed engraftment following ASCT. However, in our study, the day to neutrophil engraftment post-transplantation was comparable to Ye’s EAP protocol (median: 10 vs. 10 days) (34), and consistent with other reports (16, 24, 35). Several studies have reported that higher number of infused CD34+ cells was associated with faster hematologic recovery (30, 36). We observed faster neutrophil (median days: 10 vs. 11, *p*=0.034) and platelet engraftment (median days: 11 vs. 12, *p*=0.037) in lymphoma patients who achieved optimal apheresis, while no significant difference was found in the PM subgroup(*p>*0.05). Also,the cost of EA regimen,which includes not only the financial cost but also the logistical challenges, such as the need for hospitalization, needs further exploration. A prospective randomized study comparing these costs and logistics to alternative mobilization agents like plerixafor would give us a better view.

## 5 Conclusions

In summary, this study first explored the mobilization efficacy of EA in a large cohort of r/r lymphoma patients. Our findings indicate that EA plus G-CSF is an effective and tolerable PBSC mobilization regimen for patients with r/r lymphoma, including those predicted to be poor mobilizers. This regimen could be an option for patients with r/r lymphoma, particularly those undergoing mobilization for salvage ASCT therapy while plerixafor is limited available. However, further evaluation through prospective randomized studies and a moderate reduction in EA dosages for patients at high risk of infection, such as those of advanced age or with a history of severe infections is warranted to compare the cost-effectiveness and safety profile of EA with common mobilization protocols.

## Data availability statement

The raw data supporting the conclusions of this article will be made available by the authors, without undue reservation.

## Ethics statement

The studies involving humans were approved by the ethics committee of Union Hospital, Fujian Medical University. The studies were conducted in accordance with the local legislation and institutional requirements. Written informed consent for participation in this study was provided by the participants’ legal guardians/next of kin.

## Author contributions

ZZ: Conceptualization, Data curation, Formal analysis, Funding acquisition, Investigation, Methodology, Project administration, Writing – original draft, Writing – review & editing. XL: Conceptualization, Data curation, Formal analysis, Investigation, Methodology, Project administration, Writing – review & editing, Writing – original draft. XY: Formal analysis, Investigation, Project administration, Writing – review & editing. XC: Formal analysis, Investigation, Project administration, Writing – review & editing. TL: Data curation, Formal analysis, Methodology, Writing – review & editing. XG: Data curation, Formal analysis, Methodology, Writing – review & editing. NL: Conceptualization, Formal analysis, Funding acquisition, Project administration, Resources, Supervision, Writing – review & editing.
